# Usefulness of three-phase bone scintigraphy and SPECT/CT for the diagnosis of bone lesions of systemic sarcoidosis

**Published:** 2014

**Authors:** Shigeaki Higashiyama, Joji Kawabe, Atsushi Yoshida, Kohei Kotani, Susumu Shiomi

**Affiliations:** 1 Department of Nuclear Medicine, Graduate School of Medicine, Osaka City University, Osaka, Japan

**Keywords:** Sarcoidosis, SPECT/CT, Tc-99m HMDP, Three-phase bone scintigraphy

## Abstract

We report a three-phase bone scintigraphy for the diagnosis of a peripheral bone lesion caused by systemic sarcoidosis. A 32-year-old man with suspected osteomyelitis of the right forefinger underwent three-phase bone scintigraphy with Tc-99m hydroxymethylene diphosphonate (HMDP) and single-photon emission computed tomography/computed tomography (SPECT/CT). The lesion was rich in blood flow according to flow study and blood pool study on bone scintigraphy, and was associated with an osteolytic change on SPECT/CT imaging performed 3 hours after injection of a radioisotope (RI). Whole-body bone scintigraphy indicated multiple high levels of abnormal RI accumulation. The findings of the three-phase bone scintigraphy and SPECT/CT suggested the presence of systemic sarcoidosis; however, a subsequent ^18^F-fluorodeoxyglucose positron emission tomography/CT (FDG-PET/CT) could not exclude the possibility of multiple metastases from testicular tumors. Therefore, testicular enucleation was performed, and the pathological examination confirmed the presence of sarcoidosis.

## Introduction

Sarcoidosis, characterized by the development of granulomatous inflammation, is caused by an unknown immunologic disorder that involves various organ systems, including the lung and hilar lymph nodes, gastrointestinal tract, pelvis, muscles, and occasionally the eye (1). Although asymptomatic muscle involvement occurs in 50% to 80% of patients, symptomatic involvement is very infrequent (1). Several studies have documented the usefulness of gallium-67 citrate scintigraphy and bone scintigraphy for diagnosing sarcoidosis lesions (2, 3). However, only a few studies have reported the usefulness of three-phase bone scintigraphy and single-photon emission computed tomography/computed tomography (SPECT/CT) for the diagnosis of sarcoidosis. Few reports have described the use of ^18^F- fluorodeoxyglucose (FDG) positron emission tomography/computed tomography (PET/CT) for the diagnosis of sarcoidosis; moreover, some reports have also indicated the difficulty associated with the differentiation between a neoplastic lesion and an inflammatory lesion by activity, using the standardized uptake value (SUV), which is an index of carbohydrate metabolism (4, 5). In the present report, we describe the usefulness of three-phase bone scintigraphy and SPECT/CT in the diagnosis of a patient with a bone lesion associated with systemic sarcoidosis.

## Case Report

**Figure 1:**
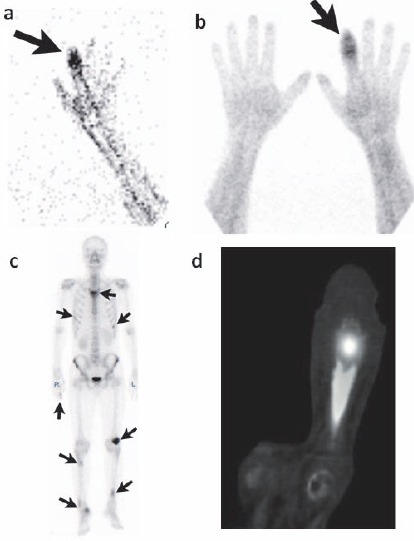
The flow **(a)** and the blood pool **(b)** studies of the three-phase bone scintigraphy. Abnormal RI accumulation was noted at the right forefinger tip. Abnormal RI accumulation in the right forefinger, sternum, bilateral ribs, left patella, bilateral tibias, and right calcaneal bone were shown on whole-body bone scintigraphy **(c)**. SPECT/CT showed the coarseness of the trabecula of the distal phalanx and abnormal RI accumulation **(d)**.

**Figure 2:**
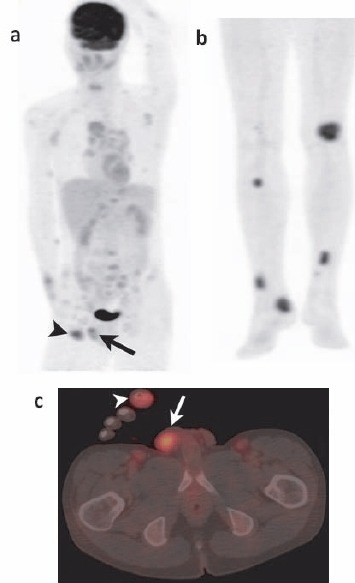
Maximum intensity projection (MIP) image of FDG-PET/CT) **(a, b)**. Abnormal FDG accumulation of multiple lymph nodes and skeletal lesions concordant with bone scintigraphy. FDG-PET/CT showed abnormal FDG accumulation on right testis (arrow) and right forefinger (arrow head) **(c)**. Bone destruction of the distal phalanx of the right forefinger was found.

A 32-year-old man presented to our institution with swelling and pain in the right forefinger. The patient was suspected of having osteomyelitis. A simple X-ray radiography showed the reticular pattern of distal phalanx of the right forefinger. Three-phase bone scintigraphy and SPECT/CT along with technetium Tc-99m hydroxymethylene diphosphonate (HMDP) bone scintigraphy were performed on the right forefinger. We administered Tc-99m HMDP (740 MBq) intravenously via a left elbow vein along with a bolus injection of 10 mL of saline. We obtained a flow study image of the right hand immediately and and a blood pool image performed 20 minutes after administration. Abnormal radioisotope (RI) accumulation was noted at the right forefinger tip on the flow study (Figure 1a, arrow) and the blood pool study (Figure 1b, arrow), indicating that the lesion was rich in blood flow. Although these findings did not contradict the possibility that the lesion was caused by osteomyelitis, a whole body bone scintigraphy examination performed after 3 hours showed high levels of abnormal RI accumulation in the right forefinger, sternum, bilateral ribs, left patella, bilateral tibias, and right calcaneal bone (Figure 1c, arrows). SPECT/CT imaging of the right forefinger indicated that the trabecula of the distal phalanx was coarse, and an osteolytic change and mass formation were evident (Figure 1d). Although osteomyelitis was not contradicted on three phase bone scintigraphy and SPECT/CT imaging, it could not be confirmed because multiple lesions were noted on imaging. As the lesion was osteolytic and rich in blood flow, a diagnosis of sarcoidosis was suspected. However, to exclude the possibility of multiple bone metastases, the chief physician suggested that FDG-PET/CT should be performed in order to identify the primary lesion. Whole body images were obtained 50 minutes after the intravenous injection of FDG (185 MBq). The multiple lesions detected on bone scintigraphy indicated abnormal accumulation of FDG on the FDG-PET maximum intensity projection image (Figure 2a, b). In addition to abnormal FDG accumulation, bone destruction of the distal phalanx of the right forefinger was noted (Figure 2 c, arrow-head). The maximum SUV of the lesion was 4.69. Multiple lymph nodes with swelling and abnormal FDG accumulation were observed (Figure 2a). Moreover, the right testis indicated abnormal FDG accumulation with a SUV of 3.9 (Figure 2a.c, arrow). As the possibility of multiple bone metastases and multiple lymph node metastases from testicular tumors could not be excluded, right testis enucleation was performed.

**Figure 3:**
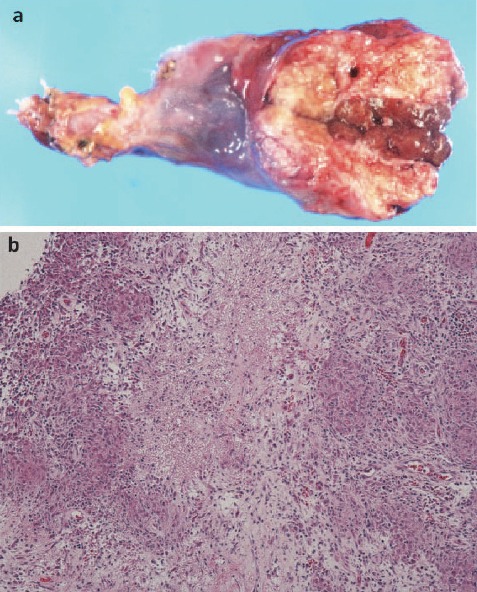
The operative histopathological specimen showed the swelling of the testes **(a)**. Histopathological study on hematoxylin and eosin staining reveal mixed granuloma tissue with epithelioid cells and multinucleated cells **(b)**.

For the surgery, an incision was made at the right inguinal ligament. An external inguinal ring was exposed, and the funiculus spermaticus was identified. The testes were exfoliated from the surrounding tissue, and the inguinal canal was opened. The funiculus spermaticus was exfoliated from the surrounding tissue. En bloc resection of the funiculus spermaticus and the testes was performed (Figure 3a). The funiculus spermaticus was found to be strongly attached to the surrounding tissue and muscular fasciae. Pathological examination confirmed the diagnosis of systemic sarcoidosis. The epithelioid granuloma comprised of mixed granuloma tissue with epithelioid cells and multinucleated cells (Figure 3b). No caseous necrosis was observed.

## Discussion

Sarcoidosis is a granulomatous disorder that mainly affects the lungs and the mediastinal and hilar lymph nodes. Multisystem involvement of the liver (70%), spleen (50%), skin (10%), salivary glands (5%) and bone (1-13%) is also a common manifestation (1, 6). The invasion of the sarcoidosis lesion to a generative organ is rare, and it can be discovered as an epididymal mass, but there are fewer testicular lesions (6). Muscular involvement of sarcoidosis presents a variable picture and is often asymptomatic. Asymptomatic muscle involvement occurs in 50% to 80% of patients, and symptomatic involvement is rare (occurring in only 0.5% of patients) (8-10). The usefulness of bone scintigraphy, gallium scintigraphy, and FDG-PET/CT has been described for the detection of sarcoidosis lesions and for the assessment of the effect of treatment (2, 3, 5). The laboratory procedure of bone scintigraphy usually involves three phase bone scintigraphy that can symptomatic the presence or absence of blood flow in the lesion. Many researchers have reported the usefulness of three phase bone scintigraphy for diagnosis of osteomyelitis (11). A previous study has indicated that three phase bone scintigraphy can detect a muscle lesion that is associated with sarcoidosis (12). However, to our knowledge, there is no published report for the detection of a bone lesion associated with Sarcoidosis. We believe that abnormal RI accumulation was found on the flow and blood pool studies because the sarcoid lesion was rich in blood flow. Three phase bone scintigraphy can evaluate the blood flow of a lesion, and an osteoblastic change can be detected by using SPECT/CT imaging. The detection of systemic lesions is a known advantage of using bone scintigraphy. In the detection of the bone lesions such as sclerosing bone metastases, the bone scintigraphy is more useful than FDG-PET (13). In the present case, we believe that the patient could have been diagnosed with sarcoidosis without the need for the invasive testicular enucleation. Three phase bone scintigraphy and SPECT/CT are useful for obtaining information on the form of and metabolism within lesions. In the present case, we used three phase bone scintigraphy and SPECT/CT to examine a patient with suspected osteomyelitis. The results of the testicular biopsy performed eventually confirmed the diagnosis of sarcoidosis that was suggested by the findings of the imaging. Three phase bone scintigraphy and SPECT/CT are useful examinations, and we believe that the presence of sarcoidosis should be considered in cases involving a blood flow–rich osteolytic lesion and the biopsy site should be carefully chosen.
